# Correction of thumb angulations after physiolysis of delta phalanges in a child with Rubinstein–Taybi syndrome: a case report

**DOI:** 10.3109/23320885.2014.997236

**Published:** 2015-01-06

**Authors:** Kousuke Iba, Takuro Wada, Toshihiko Yamashita

**Affiliations:** ^a^ Departments of Orthopaedic Surgery, Sapporo Medical University School of Medicine, Sapporo, Japan

**Keywords:** Delta phalanx, physiolysis, free-fat graft, thumb, angular deformity, correction, Rubinstein–Taybi syndrome

## Abstract

Resection of the midzone of the continuous epiphysis of a delta phalanx (physiolysis) and its replacement by a free-fat graft from local tissue was performed to improve severe radial angulation of the both thumbs in a 22-month-old child with Rubinstein–Taybi syndrome. Fifty-two months after surgery, satisfactory results were obtained for pinch function, appearance, and radiographic findings. Previous reports have indicated that corrective osteotomy of the thumbs in Rubinstein–Taybi syndrome has an associated risk of incomplete correction and >30% of recurrence, because severe preoperative deformities of the delta phalanx make angulatory osteotomies difficult. Additionally, physiolysis is a less invasive procedure than corrective osteotomy and offers a reduced risk of incomplete or excess correction, physeal injury, or osteonecrosis in younger children. Therefore, physiolysis appears to be useful as an initial means of correcting severe thumb angulations secondary to a delta phalanx. We reported a case in which the physiolysis of the delta phalanx significantly improved severe angular deformities of the thumbs in association with Rubinstein–Taybi syndrome.

## Introduction

A delta phalanx is characterized as an abnormal longitudinal physeal plate. The angular deformity of the digit due to the delta phalanx leads to impaired pinch function, which is indicative for surgical correction of the delta phalanx. However, corrective osteotomy is technically challenging in skeletally immature patients due to the risk of incomplete or excess correction, physeal injury, or osteonecrosis, especially in young children [1]. An alternative to corrective osteotomy is a limited excision at the midzone of the continuous epiphysis of longitudinal epiphyseal bracket together with physis (physiolysis) and its replacement with a free-fat graft to correct the deviation of the delta phalanx [2, 3].

Rubinstein–Taybi syndrome is a rare condition associated with severe radial angulation of the thumb, secondary to a delta proximal phalanx [4]. The angular deformities of the thumbs lead to impairment of pinch function from early childhood. Pinch function is the most important function in the successful performance of daily activities of the hands in children. However, there have been few reports on the management of thumb deformities due to delta phalanges associated with Rubinstein–Taybi syndrome [5]. In this report, the authors demonstrate that physiolysis for the delta phalanx was effective in improving the severe radial angulations of the thumbs in a patient with Rubinstein–Taybi syndrome over a 52-month follow-up period.

## Case report

A 22-month-old child was referred to us with radial angulations of the bilateral thumbs and impaired pinch function (Figures 1*a* and *b*) associated with Rubinstein–Taybi syndrome. Radiographic findings showed proximal delta phalanges and severe radial angulation at the interphalangeal joints (Figures 1*c* and *d*). The surgical procedure was performed as previously reported [2]. Briefly, the patient was given a general anesthetic and an upper arm tourniquet was applied. A mild-curved incision was made on the dorsal side of the thumb. The periosteum and some soft tissue adjacent to the incision were reflected with a scalpel to expose the area of the phalanx. Resection of the midzone of the continuous epiphysis along with the underlying physis was then performed. The cavity was filled with a free-fat graft from local tissue in the thumb (Figure 2). The periosteum and some soft tissue were then sewn over the graft to keep it in close contact with the underlying bone and cartilage. After surgery, both thumbs were fixed temporarily (2 weeks) with a Kirschner wire to stabilize the surgical site. Postoperative follow up and radiograph examination was performed at 2 weeks (at the removal of the sutures and Kirschner wire), 6 weeks and then every 3 to 6 months until 1 year. Thereafter, follow up was performed once every year. Fifty-two months after surgery, satisfactory results were obtained for pinch function and appearance (Figures 3*a* and *b*). The parents were also very satisfied with the function and appearance. According to radiographic measurements, the radial angular deformity was markedly improved in both thumbs, although some deformity remained (Figures 3*c* and *d*).

**Figure 1.**
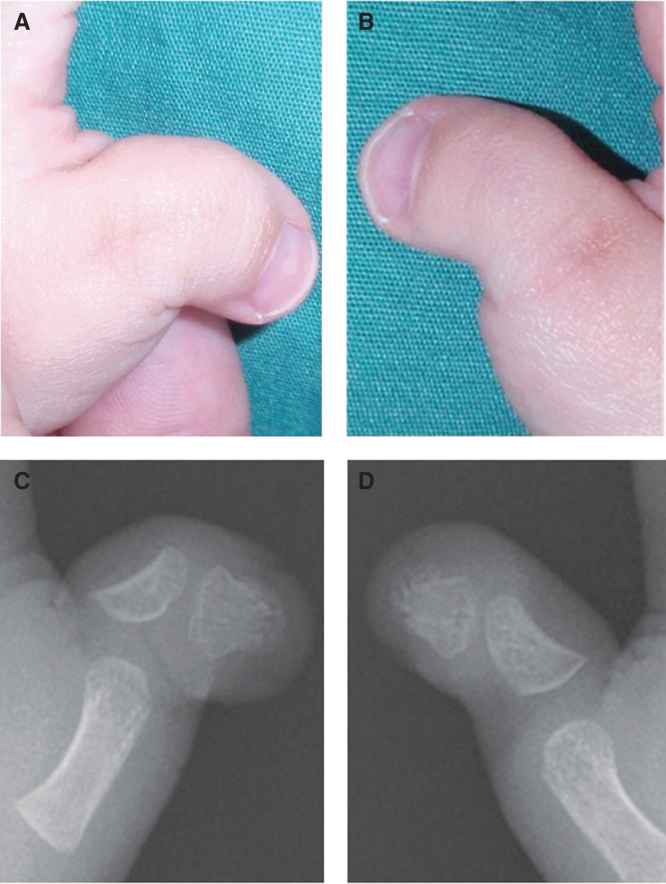
**Illustrations showing preoperative findings. The patient showed impaired pinch function due to severe radial angulations of both thumbs (*a*, left; *b*, right). Radiographic images showing proximal delta phalanges in both thumbs (C, left; D, right). The lateral deviation angle^3^, which is defined as the angle between the longitudinal axis of the metacarpal bone and that of the distal phalanx, was 78° for the left (*c*) and 54° for the right thumb (*d*).**

**Figure 2.**
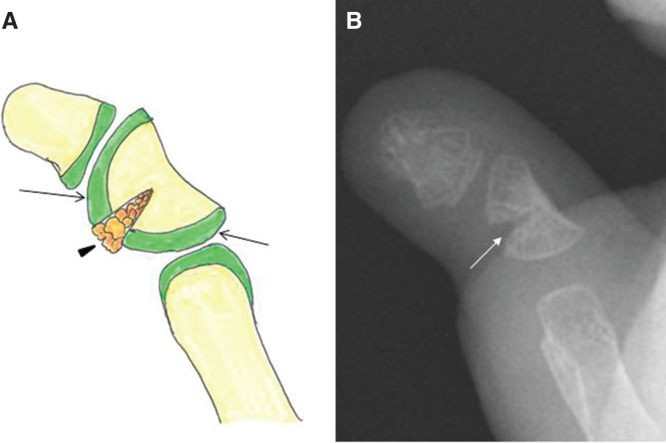
**Illustrations showing physiolysis of the delta phalanges. Resection of the midzone of the continuous epiphysis along with the underlying physis (*a*, arrow) and its replacement by a free-fat graft from local tissue (*a*, arrow head) was performed (*b*, white arrow).**

**Figure 3.**
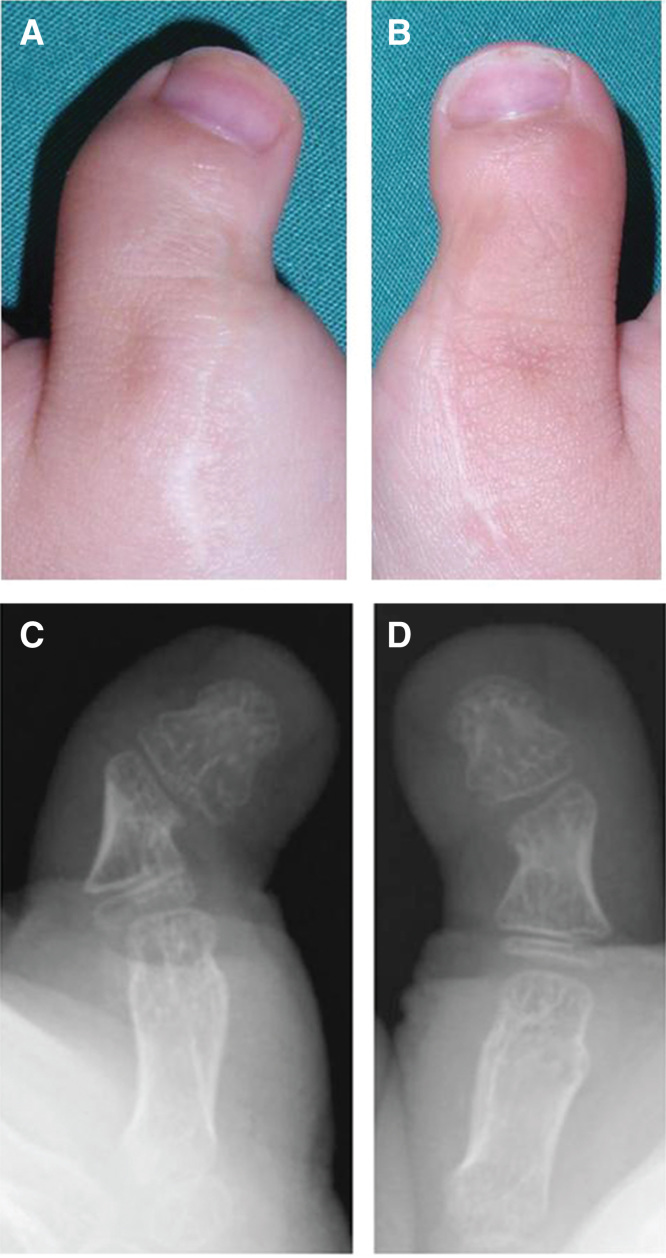
**Illustrations showing postoperative findings at 52 months after surgery. The radial angulations of the thumbs were markedly improved (*a*, left; *b*, right). According to the radiographic findings at 52 months after surgery, the lateral deviation angle^3^ was 44° for the left (*c*) and 30° for the right thumb (*d*), which indicated a correction of 34° (*c*) and 24° (*d*), respectively.**

## Discussion

Previous reports have indicated that corrective osteotomy of the thumbs could be effective in Rubinstein–Taybi syndrome patients, although the risk of recurrence due to technical issues associated with the incomplete correction of the deformity during surgery remained, with the recurrence rate reported to be >30% [5]. Jain *et al*. [5]. indicated that severe preoperative deformities in terms of the angle of the delta phalanx make angulatory osteotomies difficult, and the age of the patient at the time of surgery is important as the smaller delta phalanges observed in young children adds to the technical difficulty. They suggested that corrective osteotomy should be deferred until after the child is three-and-a-half years of age. However, angulation of the thumb prevents pinch function, which results in a reduction in fine manipulative skills [1]. Pinch function, in particularly, is the most important function for hand skill development in cases with congenital anomalies. Therefore, we think that surgical correction of the delta phalanx should be indicated when the angular deformity interferes with pinch, even in cases in which the patient is younger than three-and-a-half years. In addition, physiolysis is a less invasive procedure than corrective osteotomy and offers a reduced risk of incomplete or excess correction, physeal injury, or osteonecrosis [1, 3, 5]. Previous studies have recommended a free-fat graft to prevent fusion across the epiphyseal plate [1, 2]. In this case, we obtained sufficient fat locally in the thumb to fill the cavity without any complications.

On the other hand, we recognize some limitations to this surgical procedure. First, the age at surgery is an important factor affecting the degree of correction as better results have been observed for children who have had the surgery before 6 years of age [1, 6]. Second, a long-term follow up is required to demonstrate the effectiveness of this procedure. Third, some cases have been reported in which the correction is not complete, although pinch or grip function is markedly improved after surgery. In a few such cases, corrective osteotomy is later required.

In conclusion, we believe that this surgical method is a simple and safe procedure that is useful as an initial means of correcting severe thumb angulations secondary to a delta phalanx in congenital hand anomalies even in young children.
